# Acute physiological and pupillary responses during power snatch and clean & jerk training sessions in elite female weightlifters

**DOI:** 10.1038/s41598-026-44315-9

**Published:** 2026-03-13

**Authors:** Bülent Işık, Derviş Daşdelen, Erkan Özbay, Usame Ömer Osmanoğlu, Kenan Erdağı

**Affiliations:** 1 Departments of Physiology, Medical School, University of Karamanoğlu Mehmetbey, Karaman, 70200 Turkey; 2 Karamanoğlu Mehmetbey University Vocational School of Health, Karaman, 70200 Turkey; 3 Departments of Biostatistics, Medical School, University of Karamanoğlu Mehmetbey, Karaman, 70200 Turkey; 4 Departments of Physical Education and Sports, Ahmet Keleşoğlu Faculty of Education, Necmettin Erbakan University, Konya, 42090 Turkey

**Keywords:** Olympic weightlifting, Performing athlete health, Hemodynamic response, Physiological monitoring, Pupil diameter, Motility, Neuronal physiology, Neuroscience, Neurophysiology

## Abstract

Individual training programs are essential for healthy weightlifting. To investigate the adaptive response of vital functions and pupil diameter to olympic style weightlifting training performed in elite female weightlifters. The study was conducted with twenty elite female weightlifters in the preparation period for competitions. Weightlifters were given 90-minute training sessions with 75% and 100% maximum weight loaded on two different days. Systolic and diastolic blood pressure, oxygen saturation, pulse, respiratory rate, body temperature, and mean pupil diameter values, were measured during the rest, power snatch, clean & jerk movement and cool down phases of 75% and 100% maximum weight loaded training. Shapiro Wilk test, Friedman’s two-way analysis and Sperman correlation used for statistical analyses (*P* < 0.05). All vital values were significantly different in at least one measurement time from the other measurement times in groups (*p* ≤ 0.001). In the power snatch phase, all the vital signs were different from the rest and cool down phases in both training groups (*p* < 0.001, respectively). In 100% maximum weight training group, there was a significant positive correlation between max clean & jerk and systolic blood pressure of basal (*r* = 0.37), power snatch phase (*r* = 0.27) and cool down phase (*r* = 0.44). It can be said that different maximum weight power snatch and clean & jerk weightlifting trainings affect vital functions and mean pupil diameter changes and weightlifting performance within physiological limits, in weightlifters, and this may be a reference in arranging a training program. Continuous monitoring of athletes’ training-induced autonomic outcomes may contribute to individual arrangements for safe sports and high performance.

## Introduction

Olympic style weightlifting is a power sport in which weight is lifted using snatch, and clean & jerk techniques^1^. For a successful weightlifting performance; it is necessary to apply the highest level of muscle strength and a precise balance of power with both physical and mental fitness within the given time. High success can be gained by implementing a well-planned, balanced training program in appropriate structural, physiological and anthropometric conditions^2^. Weightlifting trainings are individual resistance training performed with specific training methods such as snatch, shouldering and throwing movements^3,4^. In addition, it has been stated that auxiliary exercises such as power snatch, power clean & jerk, back squat, front squat, power jerk behind neck, snatch balance, drop snatch, power jerk behind neck + overhead squat increase weightlifting performance, muscle strength parameters and power parameters of athletes^1,5^. The role of professional weightlifting performing athletes in being healthy and successful by implementing training programs suitable for their bodies in safe gyms is indisputable.

The physical movement axis of the muscles during sports and the dominant metabolic muscle activity during the training phases vary depending on the type, intensity and duration of the training. It is not possible to clearly distinguish static and dynamic exercise components in sports disciplines. All sports disciplines mostly include both static and dynamic exercise components together. The olympic style weightlifting is a sports branch that has primarily static and secondary dynamic exercise components, depending on the physical movement axis of the muscles during training. Moreover, in sports where most high-intensity static exercise components are dominant, such as weightlifting, gymnastics and shot put, the anaerobic metabolic pathway needs to be active in the muscles, while the aerobic metabolic pathway needs to be active in high-intensity dynamic exercises lasting more than a few minutes. The main determining parameter in the development of the weightlifter’s cardiovascular adaptation response to exercise is the pressure load. In fact, depending on the exercise components in all sports disciplines, the metabolic activity in the muscles is classified as predominantly anaerobic or aerobic according to its rate of onset. That is, all types of sports mostly involve both anaerobic and aerobic metabolic energy components that accelerate successively. The distinction is made based on the primary acceleration of the metabolic pathway that will operate to meet the energy and oxygen demand of the muscles and tissues working under the effect of exercise^6^.

When applying a training program, data regarding the percentage of VO_2max_ used, heart rate, blood lactate level and duration are often used as reference to determine the duration and intensity of the exercise to be carried out^7^. Graded exercise testing (GXT) is considered the gold standard method to determine maximum heart rate. However, this system is not always available due to limited access to the necessary equipment and qualified personnel for measurement and contraindications due to possible problematic health conditions^8^.

The formula established by Fox in 1971 (220-age) is still used for age-based maximal heart rate estimation to determine aerobic (endurance) exercise intensity^8^. However, this formula has become controversial because its overestimation of heart rate more than the formulation described by Tanaka in 2001 (208 − 0.7 x age). Therefore, in recent years, the Tanaka formula is more preferred^8–12^.

In determining the aerobic exercise intensity to be applied, the target heart rate is set to be 60–90% of the maximum heart rate. On the other hand, methods for determining anaerobic (strength) exercise intensity include one repetition maximum (1RM) or 10 repetition maximum (10RM) methods. It is recommended by the American College of Sports Medicine (ACSM) that the intensity of resistance exercise in athletes’ strength training should be 60–100% of the single 1RM and gradually increase the weight. In athletes, resistance exercise performed at least 4–6 days a week, covering different muscle groups and appropriate recovery, and at an average load intensity of 60–100% 1RM, can increase muscle strength and power^13,14^.

Weightlifting training and competitions are sports activities that, in addition to high metabolic costs, trigger important structural and functional cardiovascular adaptation mechanisms and may show different reactions depending on gender. The weightlifting athletes must display all their effort, experience and performance within seconds during the competition^5^. The elite weightlifters perform high-intensity resistance exercise, including snatch and clean & jerk techniques, with an IRM load of 80% or more for similar muscle groups almost every day of the week to increase muscle strength and performance^1,5,15,16^. In frequent and intense training programs in many competitive sports, including weightlifting, physiological responses that occur as an acute or chronic adaptation response to exercise often pose a great difficulty in achieving and maintaining homeostasis of the whole body. The body activates adaptation mechanisms that include many local and systemic, vascular and metabolic physiological processes in order to cope with difficulties during training^17,18^. Understanding the training-induced adaptation processes in weightlifters of all ages and genders may provide updated perspectives on reducing the risks of long or short-term physiological loading in athletes and providing a safe and healthy training environment^5,16^.

In the literature, it has been observed that the data obtained from the studies that have been conducted over many years up to the present day have not been able to fully clarify the structural, metabolic, physiological and vital adaptation responses elicited by different weightlifting training programs in weightlifting athletes. Athletes have been experiencing some difficulties in improving their performance with body-appropriate training practices. Many more multifaceted studies are needed to reveal all aspects of adaptation responses to different training in weightlifting athletes^15,19–25^.

Many hematological and physiological parameters, including vital functions such as hemoglobin oxygen saturation (SpO_2_), blood pressure, heart rate, and pupil diameter, are affected by training-related factors such as frequency, intensity, type and duration of exercise, as well as individual factors such as age and nutritional habits^26–30^.

Although the assessment of pupil diameter change is quite popular in neuroscience applications, it has been more intensively involved in exercise physiology applications in the last decade^31–33^. Pupil movements are a physiological condition regulated by a dynamic balance of activity between tonic sympathetic and parasympathetic autonomic nervous system components. When the parasympathetic nervous system is active, the sphincter muscles of the iris contract and myosis (pupil constriction) occurs. During exercise, with the activation of sympathetic nervous system, the radial (dilator) muscles of the iris are stimulated and mydriasis (pupil dilatation) occurs. In humans, pupil diameter can vary between 1.5 and 8 mm due to autonomic effects^34^. The increase in pupil diameter, which occurs as a result of activation in the sympathetic component of the autonomic nervous system and the inhibition of the parasympathetic component due to exercise, is a process related to the activation of many brain areas including neuromodulatory systems, medial profrontal cortex and Locus Coeruleus^35^. In addition, it has been reported that in exercises in which exercise intensity is gradually increased from low to moderate intensity, the intensity-dependent pupil diameter increases with the increase in heart. In the literature, it has been reported that this increase shows individual differences, causing increasing interest in the evaluation of pupil movement^27,36^. This information led us to investigate the effects of weightlifting training with different loads on pupillary motility.

The primary goal of weightlifting professionals in training is to obtain the best weightlifting performance. However, some physiological and metabolic changes occurred in athletes may negatively affect the duration and intensity of the training and athletic performance. The type and intensity of exercise applied to such athletes can change the physiological adaptation response to exercise^37^. In order to seize all the opportunities on the way to precise weightlifting performance, the weightlifters must develop training programs that will allow them to demonstrate fast, effective, accurate and healthy competition performance. Therefore, in weightlifting training, rest and recovery phases appropriate to the load are as important as loading with the right weight and the adaptation responses to this load. The present study aims to investigate the effects of two different weight loaded weightlifting training protocols on vital signs in elite female weightlifters who possess a high level of athletic background and compete at the national level in international competitions. The study population represents a specific group within both the general population and Olympic sports disciplines, due to their high level of elite performance, well-trained physical condition, and limited number resulting from their female gender. In the light of current information, this study aimed to examine the adaptation responses to 90 min of weightlifting training with 75% and 100% maximum weight loaded power snatch and power clean & jerk techniques in elite youth female performing weightlifters including pupil diameter change. Secondly this study aimed to examine the relationship between the vital functions and weightlifting performance. The findings may contribute to weightlifters’ training program organization and increase success in their weightlifting performance by providing a scientific reference and empirical root for weightlifting.

In summary, we hypothesized that 90 min of power weightlifting training with different weight loads in elite youth female performing weightlifters might reveal adaptation responses at different levels in terms of vital functions and that weightlifting performance might be affected by changes in vital functions. This study aims to contribute to the prevention of physiological stress and possible health risks that may be caused by high-intensity resistance training, as well as to the development of individual-specific evaluation and improvement strategies for the protection of performing athlete health. The findings obtained may contribute to the development of safer and more efficient training programs for performing weightlifting athletes, thus increasing performance as well as protecting the athlete’s health against factors associated with weightlifting. In addition, the development of individualized monitoring protocols that regularly assess vital signs such as blood pressure, heart rate, and pupil diameter at different phases of training may contribute to optimizing training safety and effectiveness.

## Materials and methods

### Ethical approval

The research was conducted in accordance with the Declaration of Helsinki^38^. The Institutional Review Board approved the study (Karamanoğlu Mehmetbey University Medicine Faculty Scientific Ethics Committee; approval number 05-2023/19; June 1, 2023), and written informed consent was obtained from the weightlifters. Informed consent for the publication of identifiable images in an online open-access publication was obtained from all participants whose photographs appear in the manuscript.

### Participants

20 elite female weightlifting athletes (all athletes on the national team during 2023–2024) between 18 and 21 years old (19.25 ± 1.65 years) who participated in international competitions (10% Olympic, 45% World, 45% European championships) were included in the research.

The athletes included in the research group consisted of elite level female athletes who were in the Turkish Weightlifting National Teams and had been actively doing weightlifting for at least 120 min 6 days per a week for the last 4 years. The weightlifters consisted of athletes from the Turkish Olympic Preparation Center and members of the Olympic team who were in the training camp preparing for the 2023–2024 competition season (Including Paris 2024 Olympic Games). The athletes’ initial measurement data were considered as their own baseline values. The initial measurement data of the weightlifters were considered as their own baseline values. The athletes were medically evaluated prior to the study. The athletes were questioned about whether they had any hematological, cardiovascular, respiratory, metabolic and orthopedic disorders and whether they had any surgery or health problems they had received treatment for before the study. Athletes with these disorders who did not meet the above-mentioned inclusion criteria were excluded from the study. The athletes were informed about the measurement parameters and times in the research. The measurements were performed in the gyms that the athletes were accustomed to, under sanitary conditions.

### Sample size calculation

The automatic direct method available in G*Power software was used, with a medium effect size of 0.75, the significance level of *α =* 0.05, power = 0.80^39^. The sample size obtained was calculated to be at least 16 weightlifters. In addition, 25% of participants were added to compensate for possible problem. Thus, the sample size to be studied was determined as 20 weightlifters. The limited number of participants was a significant constraint due to the fact that they were female weightlifters. Therefore, based on the power analysis, the present study, which was conducted with 20 elite female weightlifters, is not free from limitations regarding its statistical power and generalizability. Nevertheless, nearly all female weightlifters with high athletic capacity who were present at the training camp during the international competition preparation period were included in the study. As a result of the post-hoc power analysis with the primary clinical outcome obtained from these studies (MPD_time 2 of 75%_= 5.15 ± 0.46 mm; MPD_time 2 of 100%_= 5.32 ± 0.43 mm) and with 20 participiants, a medium effect size (Cohen’s d = 0.59) and a statistical power of 0.81 were achieved at a significance level of 0.05. The statistical power obtained meets the expected value^40,41^.

### Data collection tools

The weightlifting training age (years), weekly training duration (days) and medical histories of the athletes in the study were questioned and recorded. Body weight (kilogram) and height (centimeter) measurements were performed one day before the training, in their own rooms at the camp center, in the morning on an empty stomach and after defecation, with light clothing. Height measurements were performed with a Seca brand (213 portable mechanical, Germany) height meter, and body weights were measured with a Tanita (MC 580) brand bioimpedance weighing device, in accordance with the literature information. Body mass index (BMI) was calculated by dividing body weight (kg) by the square of height (m)^2^. All measurements of the athletes were performed by the same researcher. Records of athletes’ weightlifting performance maximum values in snatch and clean & jerk and training data were obtained from the official records kept by the technical director and the national team coaches during the training, with the permission received from the Turkish Weightlifting Federation officials.

### Training procedure

The study followed a paired, within-subject design where the same 20 athletes underwent both power snatch and clean & jerk training with 75% and 100% maximum weight loads on two separate days, 24 h apart. The athletes underwent power snatch and clean & jerk weightlifting training with 75% maximum weight loaded on the first day and 100% maximum weight loaded on the second day, 24 h apart. The training applied to the athletes in the morning on the measurement days was a total of 90 min, including 10 min of warm-up, 70 min of main training (30 min power snatch + 10 min rest + 30 min power clean & jerk) and 10 min of cool down phase respectively^42^. During the warm-up phase, the athletes performed static flexibility, joint mobility, stretching and balance exercises for 10 min. In the main training phase, training was performed in the form of 4 sets of maximum one-repetition lifts with 75% (first day) and 100% (second day) maximum weight loaded, including 30 min of power snatch and power clean & jerk movements, with a 10-minute rest in between. A 2-minute passive recovery period was left between the sets. During the cooling phase, the athletes were given stretching and flexibility exercises for 10 min^43^. In the study, the measurement data obtained from the participants during the resting period were considered as basal values. The study was conducted in accordance with a method in which seven distinct physiological parameters belonging to each participant were recorded at four specific time points during a 90-minute training session. For example, the variables measured during the resting period represented the participant’s basal control data. The study design is a within-subject repeated-measures approach, which reduces inter-individual variability and provides a preliminary framework for understanding physiological responses to different training intensities.

**Fig. 1:**
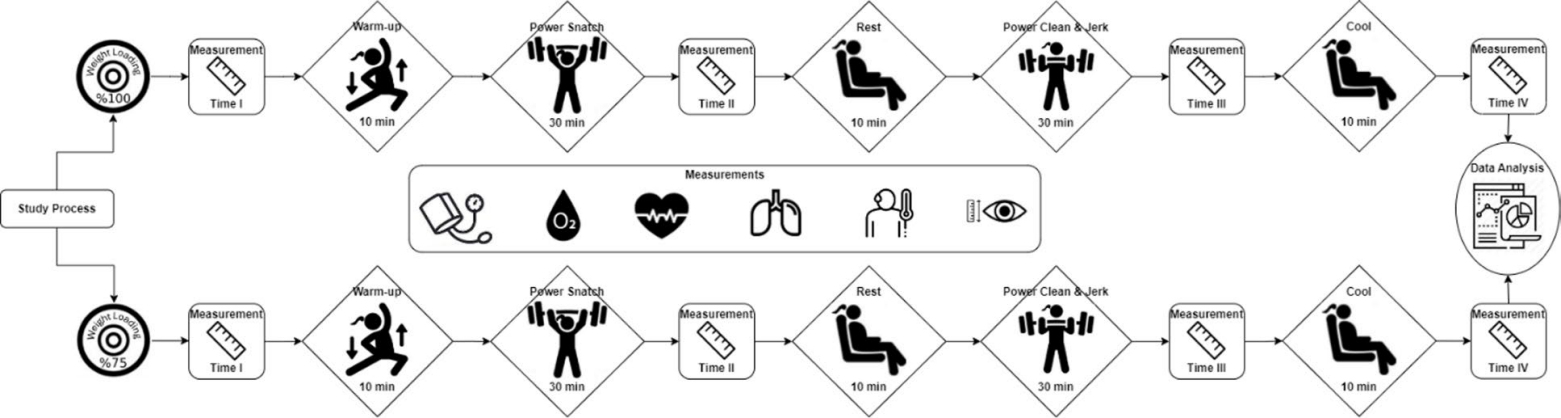
Study process.

The training program and strategies applied in the study were determined by expert weightlifting coach. Study process and measurements were performed in the following order (see Fig. 1). This protocol has been structured to observe physiological adaptation responses that may occur against high-intensity training and to obtain scientific data appropriate to the individual load-tolerance levels of performing athletes.

#### Measurement time I

During the resting phase; systolic blood pressure (SBP), diastolic blood pressure (DBP), heart rate (HR) and hemoglobin oxygen saturation (SpO_2_), body temperature (BT), respiratory rate (RR) and right and left pupil diameters were measured and the average of the pupil diameters was calculated.

Warm-up phase; 10 min.

The main phase of the power snatch movement; 30 min.

#### Measurement time II

Immediately after the main phase power snatch, SBP, DBP, HR and SpO_2_, BT, RR, MPD were measured.

Rest phase between power snatch and clean & jerk movement; 10 min.

The main phase of the power clean and jerk movement; 30 min.

#### Measurement time III

SBP, DBP, HR and SpO_2_, BT, RR, MPD were measured immediately after the main phase power clean & jerk movement.

Cooling phase; 10 min.

#### Measurement time IV

Within 5 min after the cool down phase; SBP, DBP, HR and SpO_2_, BT, RR, MPD were measured.

### Measurement of vital functions

**Fig. 2:**
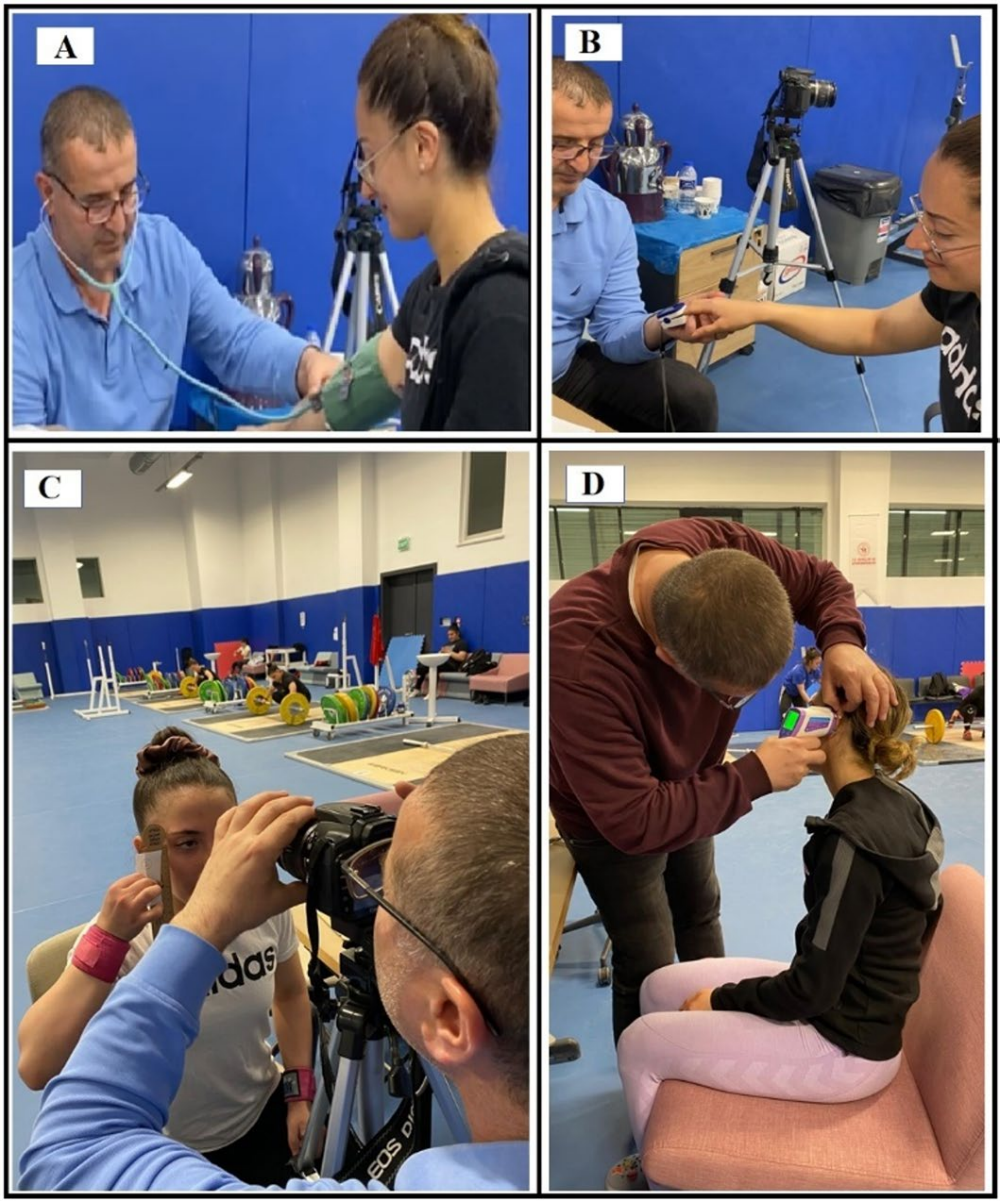
Experimental setting photos. (**A**)- Blood pressure measurement, (**B**)- Hemoglobin oxygen saturation and heart rate measurement, (**C**)- Pupil image photographing, (**D**)- Body temperature measurement.

The blood pressure of the athletes was measured twice, with an interval of 15 s, on the left arm, in a sitting position, using an Erka (Model: Perfect Aneroid, D-836446, Germany) classic arm-coil blood pressure monitor (see Fig. 2A). The averages of SBP and DBP values of these two measurements were recorded in millimeters of mercury (mmHg). HR and SpO_2_ values of the athletes were measured with a G Life Pulse Oximeter (Model: YK-81 A-Germany) in a sitting position for at least 15 s after wiping the sweaty finger and the probe (see Fig. 2B), and the results were recorded as beats/minute and percentage (%)^44^. BT measurement was performed using a tympanic infrared thermometer (Thermo-Scan, 0297) from the ear (see Fig. 2D). Since the hypothalamus (temperature regulation center of the body) and tympanic membrane most accurately reflect the core body temperature, measurements were performed through the tympanic membrane by placing the infrared thermometer in the external auditory canal and pulling the auricle upward and partially back^45^. The results were recorded in °C. RR measurement was made using a stopwatch and recorded as a number. Measurement of right and left pupil diameters was performed using an original high-resolution Canon digital fundus camera (1.5 ft, 0.45 mm) in the same room temperature (22 ± 3 °C) and appropriate illumination environment. Photography was taken at a 90º angle, 30 cm away from the center, in a similar temperature and light environment (see Fig. 2C). The right and left pupil diameters and change rates in the images obtained from the photographs were compared digitally after enlarging 4288 × 2848 pixels^46^. At the measurement time points, instantaneous photographs of the pupils were taken from a close distance and evaluated digitally on a one-to-one basis, thereby minimizing the risk of recording errors in instantaneous pupil diameter changes. Differences and change values between right (RPD) and left (LPD) pupil diameters and average pupil diameters (MPD = RPD + LPD / 2) were evaluated statistically. These physiological measurements allow the early determination of possible risks that may occur after intense resistance exercise, providing a scientific basis for protecting the health of performing athletes and adapting training programs at an individual level. Although significant controllable environmental factors such as ambient light, temperature, measurement distance, and angles were carefully regulated during pupil diameter measurements, an innovative application in weightlifting and exercise science, it cannot be stated that this method fully meets the conditions of a universal standardized continuous protocol that would ensure reproducibility across different laboratories. This has been considered one of the methodological limitations of the study^26^. A pilot study was conducted prior to the research in order to test the reliability of the method used in the study. Below are the data regarding the test-retest reliability coefficients of both the pilot study and the present study.

#### Detailed reliability data pilot study results

To ensure the test-retest reliability of the measurement parameters, all vital function clinical data (including spirometric and psychological parameters belonging to the resting period) were recorded by conducting two measurements 5 days apart in a pilot study consisting of 9 athletes before the main research. Intraclass correlation coefficient (ICC) values of the data belonging to the resting measurement period in the pilot study were calculated. To demonstrate the precision and reliability of measurements covering all vital clinic parameters, ICC values with 95% confidence intervals range from 0.90 (95%CI 0.53–0.98) to 0.99 (95%CI 0.98-1.00). These values indicate that the measurement reliability is excellent^47,48^.

#### Present study results for detailed reliability data

The test-retest reliability coefficients of the vital function measurement parameters, which include spirometric and psychological parameters for all measurement time points of the present study, were calculated and reported. To demonstrate the precision and reliability of measurements covering all vital clinic parameters, ICC values with 95% confidence intervals range from 0.91 (95%CI 0.61–0.98) to 0.99 (95%CI 0.95-1.00). These values indicate that the measurement reliability is excellent^47,48^.

### Statistical analysis

Statistical analysis was performed using IBM-SPSS 25.0 (SPSS, Chicago, IL). The variables including demographic and athletic data such as age, height, body weight, training age, BMI and weightlifting performances were calculated as mean, standard deviation and median (Q1-Q3). Shapiro-Wilk (as normality test), Friedman’s two-way analysis of variance and Spearman correlation analysis were applied. Continuous data are presented as median (Q1-Q3). Friedman’s Two-Way Analysis of Variance test was used for multiple comparisons of vital functions at the measurement times of 75% maximum weight loaded training group. Friedman’s Two-Way Analysis of Variance test was used for multiple comparisons of vital functions at the measurement times of 100% maximum weight loaded training group. In case of a significant Friedman test result, Dunn’s post-hoc test with Bonferroni adjustment was performed for pairwise comparisons to avoid inflated Type I error. Wilcoxon Signed Rank Test was used to compare the variables between the two weight-loaded groups (75% vs. 100%), as the data were obtained from a paired design involving the same subjects. Statistical test methods used in the statistical evaluation of variables are detailed in the footnotes of Tables. Non-parametric tests (Friedman and Wilcoxon Signed-Rank tests) were preferred for the analysis because the sample size was relatively small (*n* = 20) and the Shapiro-Wilk test indicated that the data for several variables did not follow a normal distribution (*p* < 0.05). Statistical significance level was taken as *p* < 0.05. ICC was calculated for test-retest reliability (2-way random-effects model, absolute agreement): reproducibility was considered to be “excellent” (*r* > 0.75), “good” (0.75 < *r* < 0.40), or “poor” (*r* < 0.40). Moreover, the 95% confidence interval (CI) for the ICC, was also calculated^47^. Correlation heat map graphs were created using the Python 3.7.9 (Delaware, USA) software program to show the correlation between variables in the 75% and 100% maximum weight loaded training groups. The interpretation of correlation coefficients was guided by the criteria proposed by Schober et al. (2018): 0.00-0.09 (no relationship), 0.10–0.39 (poor), 0.40–0.69 (moderate), 0.70–0.89 (significant), and > 0.90 (extremely high)^49^. Cohen’s d was interpreted using the following thresholds: trivial (< 0.20), small (0.20 to 0.60), moderate (0.61 to 1.20), large (1.21 to 2.0), and very large (> 2.0)^41,50,51^. The partial η² values ​​were interpreted as follows: 0.01 for small, 0.06 for medium, and 0.14 for large^41^.

## Results

Demographic variables in the findings were presented as mean±standard deviation median (Q1-Q3). The age of the athletes (*n* = 20) was 19.25 ± 1.65 years (18.00–21.00), their body weight was 65.45 ± 14.23 kg (51-74.7), their height was 162.05 ± 7.28 cm (157.25-165.75), their training age was 6.00 ± 2.05 years (5.00–7.00) and BMI values were calculated as 24.72 ± 4.10 kg/m^2^ (21.23–27.73). The averages of weightlifting performance values obtained by the athletes from their training records were recorded as 71.50 ± 12.62 kg (62.25-85.00) snatch (IRM), 91.95 ± 16.19 kg (79.25–105.00) clean & jerk (IRM).

The results of the comparison of SBP, DBP, SpO_2_, HR, RR, BT and MPD variables with the Friedman’s Two-Way Analysis of Variance test at the measurement times of 75% and 100% maximum weight loaded training are shown in Tables 1 and 2, respectively.

**Table 1:** Comparison results of vital function parameters at different measurement times of training at 75% weight loaded.

Parameters (*n* = 20)	Measurement Time				Effect Size (f)	Partial ƞ^2^	*p* value
	Time I	Time II	Time III	Time IV			
SBP (mmHg)	120.00 (110.75-123.75)	147.00 (143.25–150.50)	138.50 (135.00-142.00)	123.00 (115.00-127.50)	2.76	0.884	< 0.001***
DBP (mmHg)	68.50 (63.50–74.00)	82.50 (80.00-85.50)	80.00 (79.00–81.00)	69.00 (63.00-73.75)	2.04	0.807	< 0.001***
SpO_2_ (%)	97.00 (97.00–98.00)	98.00 (98.00–99.00)	98.00 (97.00–98.00)	97.00 (96.00–97.00)	1.16	0.574	< 0.001***
Heart Rate (beats/min)	78.50 (74.75–82.50)	150.50 (141.50-165.50)	138.00 (131.00-151.25)	88.50 (85.00–91.00)	4.45	0.952	< 0.001***
RR (number/min)	30.00 (27.50–32.00)	62.50 (55.75–69.50)	55.00 (49.25–58.75)	34.00 (31.25–36.75)	2.41	0.853	< 0.001***
BT (°C)	36.60 (36.50–36.60))	36.90 (36.80–36.90)	36.70 (36.60–36.80)	36.50 (36.43–36.60)	1.76	0.756	< 0.001***
MPD (mm)	4.23 (3.94–4.37)	5.06 (4.87–5.41)	4.90 (4.57–5.20)	4.72 (4.31–5.08)	1.60	0.718	< 0.001***

SBP: Systolic blood pressure, DBP: Diastolic blood pressure, SpO_2_: hemoglobin oxygen saturation, HR: Heart rate, RR: Respiratory rate, BT: Body Temperature, MPD: Mean pupil diameter. The parameters presented in Table 1 show the comparison of exercise-induced acute clinical changes during the resting phase (time I), main phase of the power snatch movement (time II), main phase of the power clean & jerk movement (time III), and cool down phase (time IV) in the training group that performed exercises with a 75% weight load. These findings reflect the hemodynamic and autonomic adaptation processes occurring in female weightlifters in response to short-term, high-intensity resistance exercises, highlighting the importance of considering exercise-specific individual physiological responses in training planning. The variables are presented as median (Q1-Q3), ***p ≤ 0.001, Friedman’s Two-Way Analysis of Variance was applied.

**Table 2:** Comparison results of vital function parameters at different measurement times of training at 100% weight loaded.

Parameters (*n* = 20)	Measurement Time				Effect Size (f)	Partial ƞ^2^	*p* value
	Time I	Time II	Time III	Time IV			
SBP (mmHg)	119.00 (113.00-123.50)	161.50 (156.00-165.00)	148.50 (144.25-150.75)	127.00 (123.00-131.25)	3.03	0.902	< 0.001***
DBP (mmHg)	68.00 (62.75–74.75)	86.00 (84.00-88.75)	83.00 (81.00–84.00)	72.50 (66.00–77.00)	2.34	0.846	< 0.001***
SpO_2_ (%)	97.00 (97.00–98.00)	98.00 (98.00–99.00)	98.00 (97.00–98.00)	97.00 (97.00–97.00)	1.20	0.592	< 0.001***
Heart Rate (beats/min)	79.50 (76.00–82.00)	166.50 (159.25-183.25)	150.50 (146.00-159.00)	94.00 (87.50–97.00)	5.50	0.968	< 0.001***
RR (number/min)	31.00 (28.25–32.75)	67.50 (60.00-71.75)	59.50 (53.75–62.50)	36.00 (33.25–37.75)	3.24	0.913	< 0.001***
BT (°C)	36.55 (36.40–36.70)	36.85 (36.80–36.90)	36.70 (36.60–36.80)	36.50 (36.43–36.58)	1.91	0.784	< 0.001***
MPD (mm)	4.16 (4.01–4.36)	5.33 (4.99–5.70)	4.78 (4.51–5.09)	4.39 (4.11–4.64)	2.31	0.842	< 0.001***

SBP: Systolic blood pressure, DBP: Diastolic blood pressure, SpO_2_: hemoglobin oxygen saturation, HR: Heart rate, RR: Respiratory rate, BT: Body temperature, MPD: Mean pupil diameter. The parameters presented in Table 2 show the comparison of exercise-induced acute clinical changes during the resting phase (time I), main phase of the power snatch movement (time II), main phase of the power clean & jerk movement (time III), and cool down phase (time IV) in the training group that performed exercises with a 75% weight load. These findings reflect the hemodynamic and autonomic adaptation processes occurring in female weightlifters in response to short-term, high-intensity resistance exercises, emphasizing the importance of considering exercise-specific individual physiological responses in training planning. The variables are presented as median (Q1-Q3), *** *p* ≤ 0.001, Friedman’s Two-Way Analysis of Variance was applied.

Tables 1 and 2 show a statistically significant difference in all variables at minimum one measurement time from measurements at other times (All p values < 0.001).

The results of multiple comparisons of vital functions with Friedman’s Two-Way Analysis of Variance at the measurement times of 75% and 100% maximum weight loaded training are presented in Table 3.

**Table 3:** Multiple comparison results of vital function parameters at measurement times of training at 75% and 100% weight loaded.

Measurement Time	Parameters	p value	
		Training at 75% weight load (*n* = 20)	Training at 100% weight load (*n* = 20)
Time I-II	SBP (mmHg)	< 0.001***	< 0.001***
	DBP (mmHg)	< 0.001***	< 0.001***
	SpO_2_ (%)	< 0.001***	< 0.001***
	Heart Rate(beats/min)	< 0.001***	< 0.001***
	RR (number/min)	< 0.001***	< 0.001***
	BT (°C)	< 0.001***	< 0.001***
	MPD (mm)	< 0.001***	< 0.001***
Time I-III	SBP (mmHg)	< 0.001***	< 0.001***
	DBP (mmHg)	< 0.001***	< 0.001***
	SpO_2_ (%)	0,300	1,000
	Heart Rate(beats/min)	< 0.001***	< 0.001***
	RR (number/min)	< 0.001***	< 0.001***
	BT (°C)	0.016**	0.020*
	MPD (mm)	< 0.001***	0.001***
Time I-IV	SBP (mmHg)	0.668	0.165
	DBP (mmHg)	1.000	0.518
	SpO_2_ (%)	1.000	1.000
	Heart Rate(beats/min)	0.086	0.086
	RR (number/min)	0.141	0.086
	BT (°C)	1.000	1.000
	MPD (mm)	1.000	0.300
Time II-III	SBP (mmHg)	0.086	0.042*
	DBP (mmHg)	0.165	0.086
	SpO_2_ (%)	0.193	0.011**
	Heart Rate(beats/min)	0.086	0.086
	RR (number/min)	0.072	0.086
	BT (°C)	0.029*	0.024*
	MPD (mm)	0.061	**0**,042*
Time II-IV	SBP (mmHg)	< 0.001***	< 0.001***
	DBP (mmHg)	< 0.001***	< 0.001***
	SpO_2_ (%)	< 0.001***	< 0.001***
	Heart Rate(beats/min)	< 0.001***	< 0.001***
	RR (number/min)	< 0.001***	< 0.001***
	BT (°C)	< 0.001***	< 0.001***
	MPD (mm)	< 0.001***	< 0.001***
Time III-IV	SBP (mmHg)	0.024*	0.165
	DBP (mmHg)	0.003**	0.042*
	SpO_2_ (%)	0.011**	0.455
	Heart Rate(beats/min)	0.086	0.086
	RR (number/min)	0.120	0.086
	BT (°C)	0.007**	0.011**
	MPD (mm)	0.029*	0.120

SBP: Systolic blood pressure, DBP: Diastolic blood pressure, SpO_2_: hemoglobin oxygen saturation, HR: Heart rate, RR: Respiratory rate, BT: Body temperature, MPD: Mean pupil diameter. The variables are presented as median (Q1-Q3), *** *p* ≤ 0.001, ** *p* < 0.01, * *p* < 0.05, Friedman’s Two-Way Analysis of Variance was applied. The parameters presented in Table 3 show the multiple comparison of exercise-induced acute clinical changes between the training groups performing exercises with 75% and 100% weight loads during the resting phase (time I), main phase of the power snatch movement (time II), main phase of the power clean & jerk movement (time III), and cool down phase (time IV). These findings reflect the hemodynamic and autonomic adaptation processes that occur in female weightlifters in response to short-term, high-intensity resistance exercises with different loading levels, highlighting the importance of considering exercise type-specific individual physiological responses in training planning.

In the 75% maximal weight loaded training groups SBP, DBP, SPO_2_, HR, RR, BT, MPD values were statistically significantly higher in the power snatch movement phase (measurement time II) than in the rest phase (measurement time I) (All p values < 0.001) (see Table 1).

In the 75% maximal weight loaded training groups; hemodynamic variables at measurement time II and measurement time I are shown as expressed respectively: SBP (147.0;120.0 mmHg, *p* < 0.001), DBP (82.50; 68.50 mmHg, *p* < 0.001), SpO_2_ (98.00; 97.00%, *p* < 0.001), HR (150.00; 78.50 beats/min, *p* < 0.001), RR (62.50; 30.00 number/min, *p* < 0.001), BT (36.90; 36.60 °C, *p* < 0.001), MPD (5.06; 4.23 mm, *p* < 0.001). Moreover, in the 75% maximal weight loaded training groups SBP, DBP, HR, RR, MPD values were statistically significantly higher in the power clean & jerk movement phase (measurement time III) than in the rest phase (measurement time I). In the 75% maximal weight loaded training groups; hemodynamic variables at measurement time III and measurement time I were calculated as shown respectively: SBP (138.50; 120.00 mmHg, *p* < 0.001), DBP (80.00; 68.50 mmHg, *p* < 0.001), HR (138.00; 78.50 beats/min, *p* < 0.001), RR (55.00; 30.00 number/min, *p* < 0.001), MPD (4.90; 4.23 mm, *p* < 0.001). The results are presented as in Table 1.

< Insert Table 2 >.

In the 100% maximal weight loaded training groups SBP, DBP, SPO_2_, HR, RR, BT, MPD values were statistically significantly higher in the power snatch movement phase (measurement time II) than in the rest phase (measurement time I) (All p values < 0.001) (see Table 2).

In the 100% maximal weight loaded training groups; hemodynamic variables at measurement time II and measurement time I were calculated as shown respectively: SBP (161.5; 119.0 mmHg, *p* < 0.001), DBP (86.00; 68.00 mmHg, *p* < 0.001), SpO_2_ (98.00; 97.00%, *p* < 0.001), HR (166.50; 79.50 beats/min, *p* < 0.001), RR (67.50; 31.00 number/min, *p* < 0.001), BT (36.85; 36.55 °C, *p* < 0.001), MPD (5.33; 4.16 mm, *p* < 0.001) (see Table 2). On the other hand, in the 100% maximal weight loaded training groups SBP, DBP, HR, RR, MPD values were statistically significantly higher in the power clean & jerk movement phase (measurement time III) than in the rest phase (measurement time I). In the 100% maximal weight loaded training groups; hemodynamic variables at measurement time III and measurement time I were found as indicated, respectively: SBP (148.50; 119.00 mmHg, *p* < 0.001), DBP (83.00; 68.00 mmHg, *p* < 0.001), HR (150.50; 79.50 beats/min, *p* < 0.001), RR (59.50; 31.00 number/min, *p* < 0.001), MPD (4.78; 4.16 mm, *p* < 0.001). The results are presented in Table 2.

In other words, in both training groups, SBP, DBP, HR, RR, MPD values ​​were higher in the power clean & jerk phase (measurement time III) than in the resting phase (measurement time I) (All p values < 0.001). The results are presented as in Tables 1 and 2, separately. The measurement times (multiple comparisons) where the difference occurred and p values are given in detail in Table 3. As shown in Table 3, in the 75% and 100% maximal weight loaded training groups, the BT value was statistically significantly higher in the power snatch movement phase (measurement time II) than in the power clean & jerk movement phase (measurement time III) (p values = 0.029; 0.024 respectively) (see Table 3). On the other hand, it was determined that the SBP value was statistically significantly higher in the 100% weight loaded training group in the power snatch phase (measurement time II; SBP: 161.50 mmHg) than in the power clean & jerk phase (measurement time III; SBP: 148.50 mmHg) (*p* = 0.042), (For p values results see Table 3 and for SBP values results see Table 2).

The comparison results of vital functions at the measurement times of 75% and 100% maximum weight loaded training according to weight load groups using the Wilcoxon Signed Rank Test are presented in Table 4. Comparing the groups; it was shown that the SBP (161.50; 147.00 mmHg, *p* < 0.001, d = 2.16 (indicating a very large effect)), DBP (86.00; 82.50 mmHg, *p* < 0.001, d = 1.32 (indicating a large effect)), HR (166.50; 150.50 beats/min, *p* < 0.001, d = 1.22 (indicating a large effect)), RR (67.50; 62.50 number/min, *p* = 0.013, d = 0.71 (indicating a medium effect)), and MPD (5.33; 5.06 mm, *p* = 0.012, d = 0.33 (indicating a small effect)) values of the power snatch movement (measurement time II) phase were statistically higher in the 100% maximum weight training group than in the 75% maximum weight training group (100% and 75% maximum weight training groups results are presented respectively), (see Table 4). Similarly, it was shown that the SBP (148.5; 138.5 mmHg, *p* = 0.001, d = 0.91 (indicating a medium effect)), DBP (83.00; 800.00 mmHg, *p* < 0.001, d = 1.18 (indicating a medium effect)), HR (150.50; 138.00 beats/min, *p* < 0.001, d = 0.97 (indicating a medium effect)), RR (59.50; 55.00 number/min, *p* = 0.030, d = 0.91 (indicating a medium effect)) values of the power clean & jerk movement (measurement time III) phase were statistically higher in the 100% maximum weight training group than in the 75% maximum weight loaded training group (100% and 75% maximum weight loaded training groups results are presented respectively). Moreover, the Right Pupil Diameter (4.36; 4.30 mm, *p* = 0.006, d = 0.32 (indicating a small effect)) and Left Pupil Diameter (4.38; 4.24 mm, *p* = 0.033, d = 0.34 (indicating a small effect)) values of the cooling phase (measurement time IV) were statistically significantly higher in the 100% maximum weight loaded training group (100% and 75% maximum weight loaded training groups results are presented respectively). On the other hand, it was shown that the MPD (4.72; 4.39 mm, *p* = 0.015, d = 0.25 (indicating a small effect)) value of the cooling phase (measurement time IV) was statistically higher in the 75% maximum weight training group than in the 100% maximum weight training group (75% and 100% maximum weight loaded training groups results are presented respectively). The results are presented as in Table 4.

**Table 4:** Comparison results of vital function parameters of training at 75% and 100% weight loaded at different measurement times according to group.

Measurement Time	Parameters	Training Weight Load Groups		p Value	Effect Size (d)
		75% (*n* = 20)	100% (*n* = 20)		
Measurement Time I	SBP (mmHg)	120.00 (110.75-123.75)	119.00 (113.00-123.50)	0.761	0.02
	DBP (mmHg)	68.50 (63.50–74.00)	68.00 (62.75-74.00)	0.469	0.05
	SpO_2_ (%)	97.00 (97.00–98.00)	97.00 (97.00–98.00)	0.414	0.15
	Heart Rate (beats/min)	78.50 (74.75–82.50)	79.50 (76.00–82.00)	0.288	0.08
	RR (number/min)	30.00 (28.25–32.75)	31.00 (28.25-32.00)	0.087	0.17
	BT (°C)	36.60 (36.50–36.60)	36.55 (36.40–36.70)	0.665	0.01
	MPD (mm)	4.23 (3.94–4.37)	4.16 (4.01–4.36)	0.455	0.01
	Right Pupil Diameter (mm)	4.24 (3.93–4.37)	4.17 (4.00-4.38)	0.247	0.01
	Left Pupil Diameter (mm)	4.21 (3.95–4.39)	4.18 (4.02–4.36)	0.681	0.01
Measurement Time II	SBP (mmHg)	147.00(143.25–150.50)	161.50 (156.00-165.00)	< 0.001***	2.16
	DBP (mmHg)	82.50 (80.00-85.50)	86.00 (84.00-88.75)	< 0.001***	1.32
	SpO_2_ (%)	98.00 (98.00–99.00)	98.00 (98.00–99.00)	0.317	0.17
	Heart Rate (beats/min)	150.50 (141.50-165.50)	166.50 (159.25-183.25)	< 0.001***	1.22
	RR (number/min)	62.50 (55.75–69.50)	67.50 (60-71.75)	0.013**	0.71
	BT (°C)	36.90 (36.80–36.90)	36.85 (36.80–36.90)	0.871	0.01
	MPD (mm)	5.06 (4.87–5.41)	5.33 (4.99–5.70)	0.012**	0.33
	Right Pupil Diameter(mm)	5.09 (4.86–5.45)	5.36 (4.99–5.73)	**0**,007**	0.40
	Left Pupil Diameter(mm)	5.07 (4.87–5.38)	5.27 (4.99–5.60)	0.021*	0.34
Measurement Time III	SBP (mmHg)	138.50 (135.00-142.00)	148.50 (144.25-150.75)	0.001***	0.91
	DBP (mmHg)	80.00 (79.00–81.00)	83.00 (81.00–84.00)	< 0.001***	1.17
	SpO_2_ (%)	98.00 (97.00–98.00)	98.00 (97.00–98.00)	0.589	0.02
	Heart Rate (beats/min)	138.00 (131.00-151.25)	150.50 (146.00-159.00)	< 0.001***	0.97
	RR (number/min)	55.00 (49.25–58.75)	59.50 (53.75–62.50)	0.030*	0.61
	BT (°C)	36.70 (36.60–36.80)	36.70 (36.60–36.80)	0.513	0.01
	MPD (mm)	4.90 (4.57–5.20)	4.78 (4.51–5.09)	0.794	0.20
	Right Pupil Diameter(mm)	4.91 (4.59–5.24)	4.76 (4.53–5.13)	0.765	0.12
	Left Pupil Diameter(mm)	4.89 (4.55–5.16)	4.80 (4.45–5.10)	0.681	0.11
Measurement Time IV	SBP (mmHg)	123.00 (115-127.50)	127.00 (123.00-131.25)	< 0.001***	0.74
	DBP (mmHg)	69.00 (63.00-73.75)	72.50 (66.00–77.00)	< 0.001***	1.78
	SpO_2_ (%)	97.00 (96.00–97.00)	97.00 (97.00–97.00)	0.058	0.49
	Heart Rate (beats/min)	88.50 (85.00–91.00)	94 (87.50–97.00)	< 0.001***	0.06
	RR (number/min)	34.00 (31.25–36.75)	36.00 (33.25–37.75)	0.062	0.04
	BT (°C)	36.50 (36.43–36.60)	36.50 (36.43–36.58)	0.261	0.01
	MPD (mm)	4.72 (4.31–5.08)	4.39 (4.11–4.64)	0.015**	0.25
	Right Pupil Diameter(mm)	4.30 (4.07–4.54)	4.36 (4.14–4.74)	0.006**	0.32
	Left Pupil Diameter(mm)	4.24 (3.95–4.54)	4.38 (4.13–4.67)	0.033*	0.34

SBP: Systolic blood pressure, DBP: Diastolic blood pressure, SpO_2_: hemoglobin oxygen saturation, HR: Heart rate, RR: Respiratory rate, BT: Body temperature, MPD: Mean pupil diameter. The variables are presented as median (Q1-Q3), *** *p* ≤ 0.001, ** *p* < 0.01, * *p* < 0.05, Wilcoxon Signed Rank Test was applied. The parameters presented in Table 4 demonstrate the comparison of exercise-induced acute clinical changes between the groups performing training sessions with 75% and 100% weight loads during the resting phase (time I), main phase of the power snatch movement (time II), main phase of the power clean & jerk movement (time III), and cool down phase (time IV). These findings reflect the hemodynamic and autonomic adaptation processes that occur in female weightlifters in response to short-term, high-intensity resistance exercises under different loading conditions. The observed changes associated with varying weight loads may serve as a guiding tool in determining weightlifters physiological limits and ensuring the sustainability of optimal performance levels, as well as providing insights for individualized training load management.

**Fig. 3:**
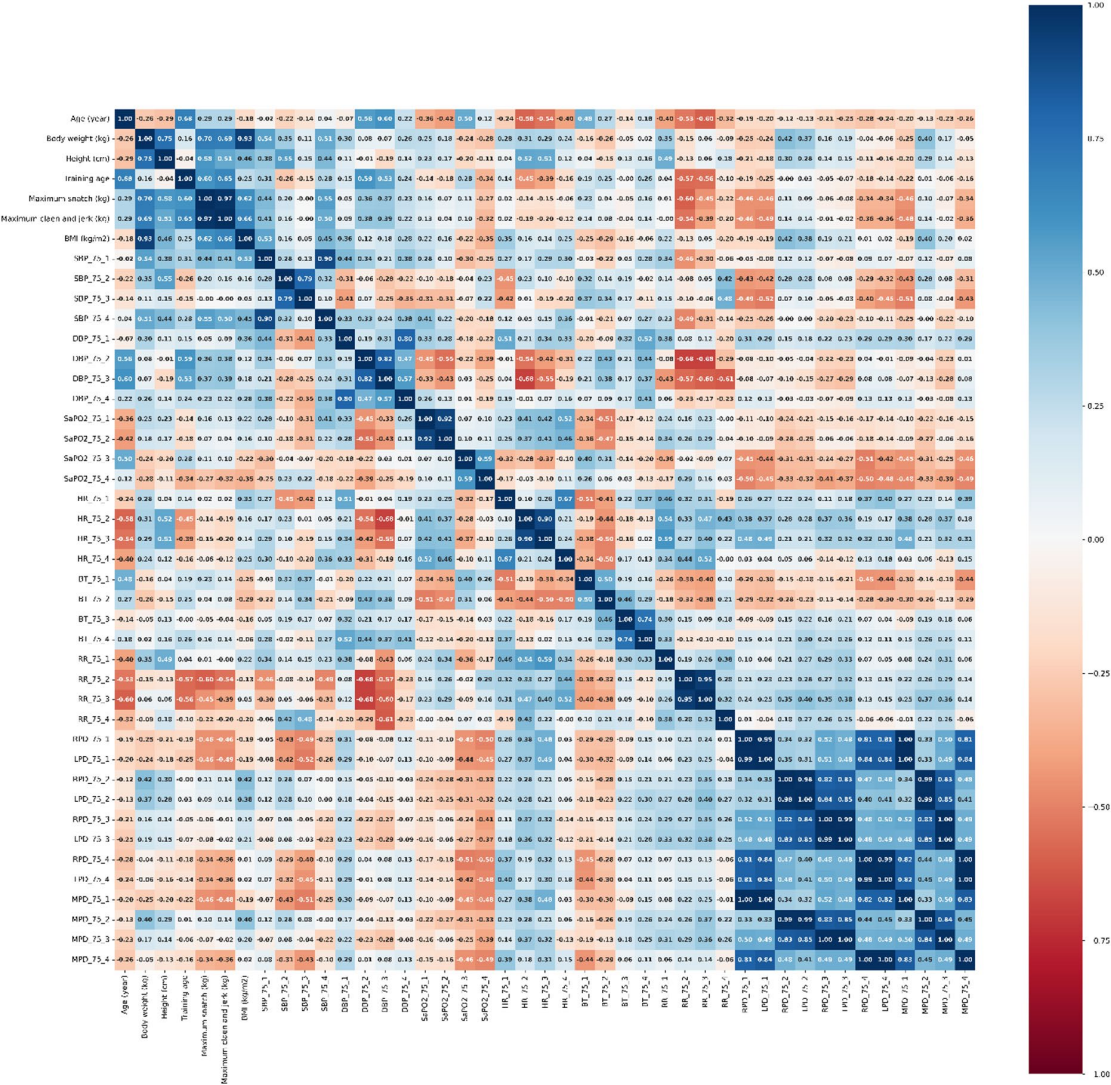
The Heat map plot for correlation in groups of training with 75% weight loaded. SBP: Systolic blood pressure, DBP: Diastolic blood pressure, SpO_2_: hemoglobin oxygen saturation, HR: Heart rate, RR: Respiratory rate, BT: Body temperature, MPD: Mean pupil diameter. Max-snatch (kg): Maximum weight lifted in snatch, Maximum clean & jerk (kg): Maximum weight lifted in clean & jerk, BMI: Body mass index.

**Fig. 4:**
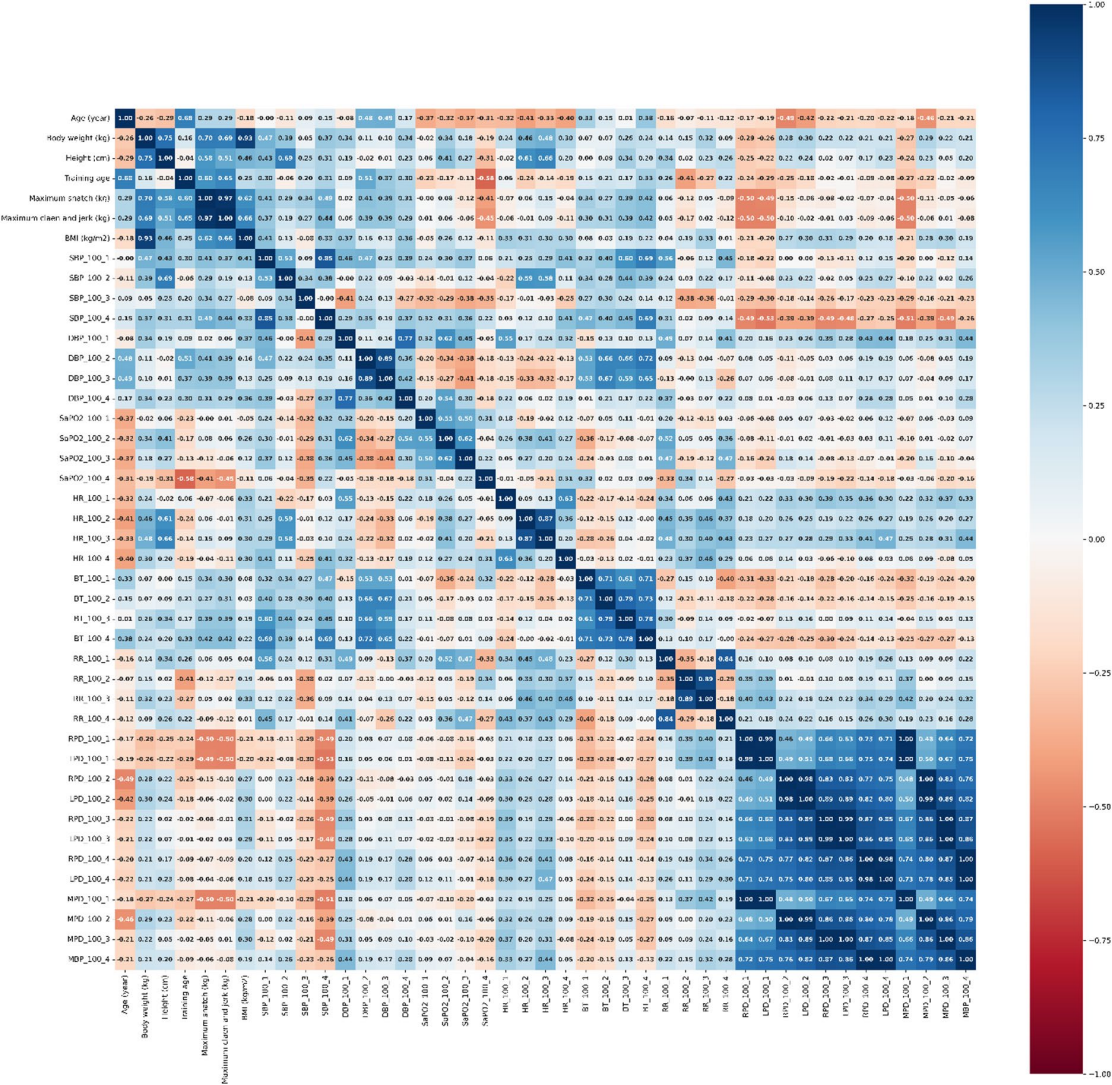
The heat map plot for correlation in groups of training with 100% weight loaded. SBP: Systolic blood pressure, DBP: Diastolic blood pressure, SpO_2_: hemoglobin oxygen saturation, HR: Heart rate, RR: Respiratory rate, BT: Body temperature, MPD: Mean pupil diameter (mm). Max-snatch (kg): Maximum weight lifted in snatch, Maximum clean & jerk (kg): Maximum weight lifted in clean & jerk, BMI: Body mass index.

Evaluation data made by Spearmen correlation analysis of vital function parameters at different measurement times in 75 and 100% maximum weight loaded training groups are presented in Figs. 3 and 4, respectively.

It was shown that BMI value was moderate positively related to weightlifting performance in snatch (*r* = 0.62, *r* = 0.66, respectively) and clean & jerk (*r* = 0.45, *r* = 0.66, respectively) in the 75% and 100% weight loaded training group. On the other hand, it has been shown that training age is moderate positively correlated with the parameters which are DBP (*r* = 0.59, *r* = 0.51, respectively), max snatch (*r* = 0.60, *r* = 0.65, respectively), and max clean & jerk (*r* = 0.60, *r* = 0.65, respectively) in both 75% and 100% maximum weight loaded training groups in the power snatch movement phase (Results are presented for the 75% and 100% maximum weight training groups, respectively), (see Fig. 3 for 75% maximum weight loaded training groups results and see Fig. 4 for 100% maximum weight loaded training groups results). On the other hand, in the 75% weight loaded training group, it was shown that there was a moderate correlation between the max snatch in resting phase SBP (*r* = 0.44), the cool down phase SBP (*r* = 0.55) value, and a slight (poor) positive correlation between the power snatch movement phase SBP (*r* = 0.20) value (see, Fig. 3). Similarly, in the 100% weight loaded training group, there was a moderate positive correlation between max clean & jerk and resting phase SBP (*r* = 0.37) and cooling phase SBP (*r* = 0.44), and a low-level positive correlation between power clean & jerk movement phase SBP (*r* = 0.27), (see, Fig. 4).

On the other hand, a moderate negative relationship (*r*=-0.50, *r*=-0.50, respectively) was shown between the resting phase MPD value and max snatch and max clean & jerk weightlifting performance values in the 100% maximum weight loaded training group (see, Fig. 4). It was determined that this negative relationship turned into a weak positive relationship between MPD in the power snatch movement phase and weightlifting performance in the 75% maximum weight training group (*r* = 0.10, *r* = 0.14, respectively), (see, Fig. 3). Additionally, it was determined that there was a moderate positive relationship between the clean & jerk movement phase MPD value and HR values in the 75% and 100% weight loaded groups (*r* = 0.32, *r* = 0.31, respectively), (Results are presented for the 75% and 100% maximum weight training groups, respectively), (see Fig. 3 for 75% maximum weight loaded training groups results and see Fig. 4 for 100% maximum weight loaded training groups results).

These findings indicate that physiological responses induced by high-intensity resistance exercises can be closely monitored, and that such data can be utilized to develop individualized training strategies aimed at protecting athlete health and enhancing performance.

##  Discussion

Important vital functions for the body include breathing, heartbeat, blood pressure, body temperature, and dynamic processes including oxygen saturation of hemoglobin in the blood and capillary fullness^52^.

In this study, the physiological effects of power snatch and power clean & jerk weightlifting training performed with different maximum weight loads on vital functions and pupil diameter change in elite female weightlifters, as well as the relationships between vital functions and weightlifting performance, were investigated. In other words, the individual physiological adaptation responses that 90 min weightlifting training with different loads produced in professional athletes and their effects on weightlifting performance were investigated. One of the main findings of the current study was that the SBP, DBP, HR, RR and MPD parameters in the power snatch movement in the main phase of the training and the SBP, DBP, HR and RR parameters in the power clean & jerk movement were significantly higher in the 100% maximum weight loaded training group than in the 75% maximum weight loaded training group. Another striking issue in the findings of our research conducted on female weightlifters with an average training age of 6 years was that SBP, DBP, HR, RR, BT and MPD parameters were significantly higher from the rest phases in the power snatch and power clean & jerk phases with in the main phases themselves in both training groups. Similarly, DBP and BT parameters were significantly higher in the main phases of training than in the cooling phase in both groups. It was observed that in weightlifting sports, where metabolically anaerobic training components are dominant, the SpO_2_ parameter did not change much with the effect of the applied training, but it was higher in the power snatch phase than the rest and cooling phases in both training groups.

On the other hand, BMI, SBP, DBP and training age parameters were positively related to snatch and clean & jerk weightlifting performance in both training groups. For instance, in the training groups with 75% and 100% weight loads, the BMI values showed a moderately positive correlation with snatch performance (*r* = 0.62 and *r* = 0.66, respectively) and clean & jerk performance (*r* = 0.45 and *r* = 0.66, respectively). This finding highlights the importance of BMI, body muscle mass, and strength parameters for weightlifters striving to achieve optimal performance (see Figs. 3 and 4)^1,2,5^.

Weightlifting, which is a resistance sport with a high static exercise component such as shot put, sprinting and jumping, is a power sport in which anaerobic energy systems are predominantly operated, but it also includes dynamic (aerobic) exercise features due to the many movements it contains^6,53^.

The adaptation response of the cardiovascular system to exercise includes many structural and metabolic changes caused by central and peripheral effects managed by the ventromedullary center. The purpose of adaptation to exercise is to provide metabolic requirements, including the energy and oxygen needs of working muscles, to reduce increased body temperature and to maintain blood flow to vital organs. This process involves increasing cardiac output as a result of increasing cardiac stroke volume and heart rate through a series of complex functions. During exercise, maximum HR control is provided via the autonomic nervous system by the motor cortical center, thus maintaining central blood pressure and tissue reperfusion^29,53^. Feed-forward back control mechanisms work in regulating cardiovascular functions during short-term exercise. When needed, in order to ensure adequate circulation, blood pressure is increased by increasing heart rate and cardiac contractility through the stimulations coming from the cortical upper center before and during exercise^29^. By suppressing the parasympathetic activity, which is active at rest, sympathetic activity becomes active with exercise. During exercise, the blood flow of the tissues is increased in proportion to the intensity and duration of the exercise. An increase in the blood flow of tissues is achieved due to vasodilatation and decrease in peripheral resistance resulting from metabolic products such as hydrogen, adenosine and carbon dioxide released in the tissues due to increased metabolic activities during exercise. With the effect of vasoconstriction in the splanchnic area, blood is transferred to vital organs and venous return increases with the pump effect of muscle movements, and as a result, cardiac output increases. Physiological changes in vital signs during exercise occur through the activation of peripheral receptors and their coordinated work with the central effect^53,54^. From a physiological standpoint, the increase in sympathetic activity that occurs to meet increased hemodynamic and metabolic needs due to visceral autonomic afferent signals from baroreceptors and chemoreceptors in blood vessels, thermoreceptors in the skin and viscera, and mechanoreceptors in the lungs under the influence of exercise triggers many outcomes. It is well established that this response may involve catecholamine release from the adrenal medulla via activation of preganglionic sympathetic pathways, as well as cardiovascular, respiratory, and metabolic adaptations mediated by adrenergic receptors. In addition, there may also be changes in central neural activity of the brain. In the autonomic control of all these hemodynamic and metabolic processes, higher neural centers such as the cortex, hypothalamus, limbic system, brainstem and spinal cord play an active role^34,54^. These mechanisms are discussed as a physiological framework supporting the observed indicators of increased sympathetic activity in response to exercise in the present study.

Although there are a limited number of studies in the literature investigating the effects of training types applied to female weightlifting athletes on vital functions, there are many studies investigating the relationship between vital functions and short-term training in sports branches other than weightlifting, where anaerobic energy metabolism is predominant. In a study by Suna and Alp (2019) in which vital functions were investigated in elite male tennis athletes during the competition period; it was found that HR and SpO_2_ parameters at rest before the competition were 94.88 ± 9.91 beats/min; 96.13 ± 1.64%, while maximum HR and SpO_2_ parameters were 157.90 ± 12.06 beats/min; 96.13 ± 2.47% during the competition^55^. The findings were similar to the HR and SpO_2_ parameters shown in the rest phase and the main phase of the clean & jerk movement of the training groups of our study. While some studies have shown a positive relationship between sympathetic activation predictor HR and match performance, similarly, our study showed a positive relationship between weightlifting performance and blood pressure parameters^56^. In another study investigating heart rate changes during competition in senior female taekwondo athletes who need anaerobic energy system such as weightlifting, the resting and main circuit HR changes of the athletes were similar to the HR findings in the training phases of our study^57^.

In the development of an adaptation response to exercise in the form of increased cardiac output, the increase in HR is ahead of the increase in cardiac stroke volume. During rest, the parasympathetic effect on HR is dominant. Low resting HR is a sign of adaptation to regular exercise that is more common in well-trained athletes^58^. It was thought that the higher HR, MPD and blood pressure parameters in the main training phases of our study groups compared to the inactive phases such as rest and cooling were due to increased sympathetic activation to meet the blood flow needs of the tissues. In other words, it can be stated that the physiological basis of the data obtained by Hayashi and Someya (2011) through the measurement of autonomic markers such as heart rate, pupil diameter, and blood pressure during resistance exercise aligns with the findings observed in the weightlifters of the present study, and that this may contribute to the evaluation of exercise-induced autonomic sympathetic activation through MPD measurement^26^. It was evaluated that the higher blood pressure parameters in our study 100% maximum weight loaded training group during the main phases of the training may be due to the intensity and intensity of the training.

Moreover, the findings of increased MPD due to the effect of weightlifting training in our study also coincide with the findings of increased pupil diameter due to increased sympathetic activity shown in studies investigating effect of isometric resistance exercise on pupil movements in female participants^59,60^.

In some studies, conducted with weightlifting athletes in the literature, it has been shown that resting HR values ranged from 60 to 82 beats/min, SBP values range from 114 to 121 mmHg, and DBP values range from 78 to 85 mmHg^61,62^. It was observed that the resting vital functions in our research conducted with female weightlifting athletes were largely similar to the information in the literature. In a study conducted on competitive male weightlifters who have been doing weightlifting for more than six years, it has been shown that the HR value was increased from 60.00 ± 9.00 beats/min to 87.00 ± 6.00 beats/min, and the SBP value was increased from 114.00 ± 7.00 mmHg to 186.00 ± 6.00 mmHg, with the effect of short-term isometric exercise^61^. The effect of exercise on vital functions was consistent with the findings of our elite female athletes. On the other hand, the findings of the present study are also consistent with those of Hayashi et al. (2010), who reported increases in heart rate, pupil diameter, and blood pressure associated with autonomic sympathetic activation during sports activities predominantly involving dynamic rather than static exercise components^36^. Similarly, the study conducted by Padulo et al. (2014), which investigated the potential effects of whole-body vibration on running and walking performance in marathon runners, provides valuable insights for comparing the acute physiological responses (such as heart rate elevation) to high-intensity exercise with those observed in weightlifting training^63^. In a study investigating the effects of 90-minute moderate intensity badminton training on hematological and physiological parameters on elite female and male para-badminton athletes, it was determined that the HR value of the athletes at rest was in the range of 60.00–70.00 beats/min and in the main phase of the training was in the range of 139.00-154.00 beats/min^64^. These findings were similar to the HR findings in the resting, power snatch and clean & jerk phases in the training groups of our study. It can be said that the small difference in HR values between athletes was related to the high dynamic, low static exercise component intensity of the badminton sport type. Regular training and increased load improve cardiovascular system capacity. The performance gained by increasing load in short-term anaerobic exercise is called anaerobic performance. Anaerobic capacity consists of the combination of anaerobic energy capacity and anaerobic power. In order to perform successfully in weightlifting sports that require short-term explosive power, it is vital to have a high anaerobic performance against fatigue. Pierce et al., in their study published in 2022, stated that the blood pressure change that develops as an adaptation response to weightlifting training might have a positive effect on anaerobic performance and weightlifting performance to certain extent^16^. In our study, it was observed that physiological parameters such as SBP, DBP and training age were positively related to snatch and clean & jerk weightlifting performance in the training groups. In addition, it may be considered that the increase in SBP caused by vasoconstriction in the arterial systems of the extremities and the increase in HR during training might contribute to the success of weightlifting performance. For example, the finding that training age was moderately and positively correlated with DBP (*r* = 0.59 and *r* = 0.51, respectively), max snatch (*r* = 0.60 and *r* = 0.65, respectively), and max clean & jerk (*r* = 0.60 and *r* = 0.65, respectively) parameters during the power snatch movement phase in both the 75% and 100% weight-loaded training groups supports our interpretation regarding the relationship between training-induced sympathetic activation outcomes and weightlifting performance (see Figs. 3 and 4). Similarly, in the 75% weight-loaded training group, the moderate correlations observed between maximum snatch performance and SBP values measured during the resting phase (*r* = 0.44) and the cool down phase (*r* = 0.55) are consistent with our findings on this subject (see Fig. 3)^5,6^.

The oxygen carrying capacity of the blood and the oxygen utilization capacity of the tissues are very important in aerobic exercises. Moreover, speed training or anaerobic exercise can interfere with endurance or aerobic exercise. Fast and vigorous exercise increases the amount of Adenosine triphosphate (ATP)-Creatin phosphate (CrP), which is an indispensable energy source in anaerobic exercise. While it is directly proportional to muscle mass and the amount of energy used during exercise in athletes, there is a negative relationship between heart-lung endurance and the risk of fatigue during exercise. If CrP supply is depleted in the formation of ATP, anoxic breakdown of glucose called “anaerobic glycolysis” occurs. The ATP-CrP system is very useful for muscle contraction activities that last between 3 and 8 s, such as weightlifting performed within 30 s^65,66^. From another perspective, the phosphagen system, which supplies immediate energy during exercise, the anaerobic energy pathway encompassing glycolysis, and the aerobic energy pathway are not independent systems operating in isolation. Both anaerobic and aerobic energy systems contribute to energy production at every stage of exercise. All energy systems function continuously and simultaneously to provide the energy required for the work performed during exercise. In other words, the ability of energy-producing pathways responsible for ATP synthesis and for replenishing the ATP pool consumed during exercise differs in terms of their rate of activation. At the onset of exercise (during the initial few minutes when energy expenditure and demand sharply increase) the anaerobic energy system, consisting of the phosphagen system and glycolysis, is activated more rapidly and predominates as the main energy source. In this way, the energy deficit resulting from the delayed activation of oxidative (aerobic) energy pathways is compensated, allowing the continuation of exercise. As exercise progresses, the contribution of aerobic energy production systems increases progressively with their accelerated activity, becoming the predominant energy source for meeting energy demands, while the contribution of the anaerobic pathway to energy supply gradually declines^54,65,67^. In the training groups in our study, the SpO_2_ parameter was found to be higher in the main phase movements of the training than in the rest and cooling phases. This condition can be interpreted as a training-induced metabolic response aimed at meeting the increased oxygen demand resulting from elevated HR, RR, metabolic activity, and sympathetic activation caused by exercise. Furthermore, it can be stated that this response represents a training-induced metabolic adaptation associated with exercise-related interactive factors such as the replacement of depleted oxygen stores in muscles and blood during the early stages of exercise, the resynthesis of expended ATP and CrP, the metabolism of accumulated blood lactate and its conversion back to glucose in the liver, and the increase in BT and metabolically active hormones following exercise^54,65,67^.

Also, this may be due to the intensive use of aerobic energy components in situations where oxygen demand was intense or the respiratory system contributing more oxygen in these phases. It was thought that the reason might be related to the oxygen needs of the tissues, their capacity to use oxygen, and physiological adaptation processes to exercise, rather than the oxygen carrying capacity of the blood.

Moreover, the MPD value measured during the cooling phase was found to be lower in the 100% weight training group than in the 75% weight training group, suggesting that the recovery process took longer in the 75% weight training group. These findings demonstrate the importance of continuous monitoring of physiological vital signs in weightlifting athletes in terms of individualized training loading management. Understanding the responses of vital functions and pupil movement to anaerobic training of different intensities can contribute to optimizing the recovery process, reducing the risk of overtraining and sustainability of performance. This perspective supports the development of evidence-based preventive strategies for long-term health of performing athlete, safety and athlete-specific medical application protocols for sports-related health impairments^23,68^. In a similar vein, Wheeler et al. (2023) reported that in patients with chronic patellar tendinopathy, the therapeutic efficacy of radial extracorporeal shockwave therapy (rESWT) was independent of treatment dose, with comparable recovery outcomes observed across different energy intensities. This observation highlights that physiological adaptation and recovery may depend more on individualized response dynamics rather than absolute load intensity, reinforcing the necessity of continuous physiological monitoring and personalized training prescriptions in high-performance athletes^69^. This perspective supports the development of evidence-based preventive strategies for the long-term health, safety, and performance sustainability of elite athletes. Regarding the recovery and return-to-sport process after a sports injury, in a study by Pipino et al. (2023) investigating the effects of 12 sessions of dry land and aquatic rehabilitation protocols applied after injured anterior cruciate ligament reconstruction, it was shown that the effects of both treatment protocols were similar in terms of pain and function. In the study, the authors stated that the aquatic rehabilitation protocol may shorten the treatment duration in the recovery and improvement process. However, they emphasized that the successful return-to-sport process after injury, in other words, the desired level of recovery and treatment process, can be affected by many physical and psychological factors such as neuromuscular, balance, proprioception impairments, kinesiophobia, perceived knee function, and pre-injury activity level^70^. Similarly, regarding the recovery and technical re-programming process of football players following surgical intervention due to anterior cruciate ligament injury, in the study by D’Onofrio et al. (2023), which investigated the effects of The Interval Kicking Program (IKP) model, applied as a neuromotor remodelling program after reconstructive surgery, on the re-programming of fundamental technical abilities, they showed that the IKP model is effective in the re-programming of technical abilities when integrated with therapeutic exercises and cardiovascular performance. In the study, the authors stated that, in the re-modulation of basic technique after surgical intervention due to anterior cruciate ligament injury in football players, in addition to individual factors such as body structure, age, sex, and training age, process-related factors such as the characteristics of the exercise applied, the therapeutic rehabilitation process, and previous injury history may also be effective. They emphasized the need for the IKP module to be individualized according to the athlete’s injury, rehabilitation process, and previous injury history^71^. In the two existing studies, the importance of the effects of different exercise modalities on various factors related to post-injury recovery and the treatment process in athletes is emphasized. From this point of view, in our study, it can be stated that factors such as training age and the intensity and duration of the applied exercises may be effective in the significant changes observed in the vital recovery responses, including SBP, DBP, and HR, to different weight-loaded weightlifting exercises administered to elite female weightlifters with different training backgrounds (see Table 4). Moreover, the continuous monitoring of vital data related to the recovery process draws attention to its importance in determining individual overtraining and physiological performance upper limits, as well as in preventing possible sports injuries.

Similarly, in a study by Frizziero et al. (2023) evaluating the effects of hormonal changes caused by the ovarian cycle on proprioception and neuromuscular control in women, it was reported that performance changes observed during athletic activities requiring high functional demands, due to differences in hormonal concentrations, could affect the risk of injury. The researchers noted that fluctuations in estrogen and progesterone levels, in particular, could lead to differences in neuromuscular control mechanisms by affecting elasticity and joint stability in the locomotor system. These findings indicate that hormonal exposure, which varies throughout the ovarian cycle, should be taken into account in the development of neuromuscular strategies aimed at preventing lower extremity injuries in female athletes^72^. In this context, individualized exercise programs and loading plans tailored to the athlete’s physiological state and hormonal phase are important for both performance optimization and injury risk reduction. Therefore, continuous monitoring of vital recovery parameters (Etc. MPD, SBP, DBP, HR), taking into account different training intensities and physiological factors (e.g., hormonal cycle phases, training age, and exercise history), is critical for determining individual overtraining thresholds, monitoring neuromuscular adaptation, and ensuring the sustainability of athlete health. This perspective contributes to the development of evidence-based preventive strategies for the long-term health and safety of high-performance athletes and the development of sport-specific medical practice protocols.

### Limitations and future research

While this study provides valuable information about the physiological changes caused by power weightlifting training in vital signs including blood pressure, SpO_2,_ heart rate, respiratory rate, body temperature and pupil diameter in elite female weightlifters, it faces several limitations. It is indicated that physiological responses in the Olympic weightlifting literature are largely addressed within the framework of narrative review studies^5,73^, and that empirical research has predominantly remained limited to cross-sectional designs conducted on small samples and specific genders^74,75^. The main limitation of this study is that it was conducted only on female weightlifters. Although the sample size of 20 elite female athletes was determined based on power analysis, it may still be insufficient to generalize the findings to the entire weightlifting population or to both sexes, as physiological responses may differ in male athletes. Secondly, the study lacked continuous and reproducible digital physiological monitoring methods, such as those capable of recording blood pressure, heart rate, and pupil diameter. Future studies should incorporate non-invasive physiological vital signs, including blood pressure, heart rate variability, pupil diameter, and other continuous measurements. In addition, it is recommended that future research be designed to include longer-term follow-up studies. Such designs may provide important insights into the time-dependent changes of physiological adaptations that develop in response to training load. Since the present study was limited to female athletes, comparative studies conducted with similar parameters in male athletes would be valuable for revealing sex-related differences in physiological responses. Thirdly, the absence of a true control group in this study and the lack of comparison with different types of exercise represent another important factor limiting the contextual interpretability of the observed physiological responses. Therefore, investigating the effects of various training models and intensities (e.g., interval training, plyometric exercises, or different resistance levels) on physiological parameters may contribute to diversifying the findings and enhancing their generalizability. Lastly, the generalize ability of the results was limited due to the focus on specific subgroups of the weightlifters. We thought that future studies on vital function changes during training and competition with more athletes in different branches, categories and genders would contribute to the exercise physiology and weightlifting literature in terms of training planning and performance improvement. In future research, it is important to develop more advanced, non-invasive and continuous monitoring physiological dynamic parameters monitoring systems that will help prevent possible health risks and overtraining situations in performing athletes. Such systems can contribute to the protection of athlete health by providing early detection of abnormal physiological responses to high-intensity exercises. Applying similar monitoring approaches in workplace-like high-performance sports environments may also contribute to the development of preventive strategies between the fields of sports science and occupational health. Finally, the integration of the vital signs evaluated in this study (e.g., systolic/diastolic blood pressure, heart rate, MPD, and SpO_2_) into individualized physiological monitoring systems emerges as a potential area for future research, particularly in terms of enabling the early detection of overtraining risks and facilitating the safer adjustment of training programs.

### Conclusion

In conclusion, these results provided support for our hypothesis. Firstly, the findings showed that power weightlifting training performed for 90 min with 75% and 100% maximum weight loaded in elite female weightlifters caused changes at the metabolic level in vital functions within physiological limits. Consistent with the study by Storey and Smith, 2012 physiological level sympathetic activation changes in vital functions as a response to training adaptation were more evident in the 100% maximum weight loaded training group in our study^5^. Second, the findings showed that weightlifting performance might be affected by changes in vital functions caused by training. Moreover, the fact that physiological responses elicited by different training intensities exhibit a dose-response relationship may be associated with the athlete’s individual physiological adaptation capacity. In this context, inter-individual variability may be influenced by factors such as training age, anthropometric characteristics, and previous training history. The findings show that monitoring vital functions can be an important tool in creating safer, individualized training programs with upper and lower limits for athletes. Implementation of such evidence-based strategies can contribute to the prevention of overtraining-related health problems, support of recovery processes, and long-term health of performing athlete and performance sustainability.

For example, one of the study’s findings, that SBP, DBP, HR, BT, and MPD parameters were significantly higher during the main training phases of power snatch and power clean & jerk compared to the resting phase in both groups, may provide important insights into the monitoring of athletes’ training-induced hemodynamic responses. Understanding the athlete’s training-induced sympathetic activation outcomes can enable the development of individualized, effective, and safe training strategies based on the athlete’s potential upper and lower limits of vital responses.

In summary, this study provides a reference for the training of female weightlifters’ physiological vital responses and individual adaptation states against weightlifting training and enriches the selection of alternative training programs for female weightlifters. In addition, instant monitoring of physiological vital responses and associating them with individual adaptation strategies may be an important reference not only for increasing performance, but also for protecting athlete health against challenging sports-related situations, reducing the risk of overload and creating long-term sustainable individual training programs. In this respect, the study findings contribute to the development of evidence-based preventive practices that support the health and safety of athletes.

### Practical applications

The findings of significantly higher SBP, DBP, HR, and RR in the 100% weight-loading training group than in the 75% weight-loading training group during the main training phases of the study, the power snatch and power C&J movement, may be important indicators for weightlifting athletes and coaches regarding training intensity, frequency, and scheduling in performance-enhancing training plans. Knowing and closely monitoring the upper and lower limits of athletes’ individual vital responses to training may guide the planning of safe individual training programs. The MPB parameter, which was found to be significantly higher in the 100% weight-loading training group than in the 75% weight-loading training group at the end of the power snatch movement phase, may be an important indicator for weightlifting coaches to monitor both visually and digitally, independent of other vital outputs, in terms of planning training intensity, frequency and time. Understanding and continuously monitoring the limits of athletes’ individual MPB response to training may be used to implement healthy and effective individual training programs. At this point, the study conducted by Raiola in 2023 on the importance of university education for physical education teachers and sport kinesiologists draws attention to the significance of education and professional development in sports science^76^. In particular, regular monitoring of physiological responses that develop in response to intensive training may facilitate the early detection of cardiovascular stress or overtraining symptoms, thereby contributing to the development of preventive approaches aimed at protecting athlete health. From another perspective, the findings of this study, that anthropometric parameters such as BMI and training age were moderately and positively correlated with weightlifting performance in both weight-loading training groups, may guide coaches in determining appropriate weight-load intensities and weight-adjustment strategies during competition preparation and training periods.

In addition, the establishment of individualized monitoring protocols that include the regular assessment of parameters such as blood pressure, heart rate, and pupil diameter at different stages of training may contribute to optimizing training safety and effectiveness. Ideally, these monitoring protocols should be integrated into athlete tracking systems capable of providing real-time feedback, allowing for instantaneous adjustments of training loads according to acute physiological stress responses.

Particularly in high-intensity weightlifting training, paying attention to recovery time, signs of overtraining, and autonomic sympathetic activity responses are crucial for enhancing performance adaptation and reducing the risk of injury.

Finally, despite the statistical evidence presented in this study, the clinical and practical significance of the training-induced physiological responses observed in female weightlifters requires further investigation, especially in terms of training monitoring, determination of load limits, and prevention of overtraining risks.

## Data Availability

The full study protocol, measurement procedures, and analysis scripts associated with this research have been made publicly available in a dedicated GitHub repository: [https://github.com/bulentisik/weightlifting-physiology-protocol](https:/github.com/bulentisik/weightlifting-physiology-protocol)All methodological files are accessible without restriction. The data that support the findings of this study are available from the corresponding author upon reasonable request.
